# Phosphorylation of Influenza A Virus Matrix Protein 1 at Threonine 108 Controls Its Multimerization State and Functional Association with the STRIPAK Complex

**DOI:** 10.1128/mbio.03231-22

**Published:** 2023-01-05

**Authors:** Lu Liu, Axel Weber, Uwe Linne, Mahmoud Shehata, Stephan Pleschka, Michael Kracht, M. Lienhard Schmitz

**Affiliations:** a Institute of Biochemistry, Justus-Liebig-University, Giessen, Germany; b Institute of Medical Virology, Justus Liebig University, Giessen, Germany; c Rudolf-Buchheim-Institute of Pharmacology, Justus-Liebig-University, Giessen, Germany; d Mass Spectrometry Facility, Department of Chemistry, Philipps University, Marburg, Germany; e Center of Scientific Excellence for Influenza Viruses, National Research Centre, Cairo, Egypt; f German Center for Infection Research, Partner Site Giessen, Giessen, Germany; University of Hong Kong; Johns Hopkins Bloomberg School of Public Health

**Keywords:** influenza A virus, kinase, signaling, phosphorylation, STRIPAK, budding

## Abstract

The influenza A virus (IAV)-encoded matrix protein 1 (M1) acts as a master regulator of virus replication and fulfills multiple structural and regulatory functions in different cell compartments. Therefore, the spatiotemporal regulation of M1 is achieved by different mechanisms, including its structural and pH-dependent flexibility, differential association with cellular factors, and posttranslational modifications. Here, we investigated the function of M1 phosphorylation at the evolutionarily conserved threonine 108 (T108) and found that its mutation to a nonphosphorylatable alanine prohibited virus replication. Absent T108, phosphorylation led to strongly increased self-association of M1 at the cell membrane and consequently prohibited its ability to enter the nucleus and to contribute to viral ribonucleoprotein nuclear export. M1 T108 phosphorylation also controls the binding affinity to the cellular STRIPAK (striatin-interacting phosphatases and kinases) complex, which contains different kinases and the phosphatase PP2A to shape phosphorylation-dependent signaling networks. IAV infection led to the redistribution of the STRIPAK scaffolding subunits STRN and STRN3 from the cell membrane to cytosolic and perinuclear clusters, where it colocalized with M1. Inactivation of the STRIPAK complex resulted in compromised M1 polymerization and IAV replication.

## INTRODUCTION

Influenza A viruses (IAVs) are enveloped negative-strand RNA viruses with segmented genomes containing eight gene segments. IAVs cause seasonal epidemics and occasional pandemics due to the high degree of genomic plasticity and continued evolution that enables them to escape host immunity and adapt to new host species (1). The IAV infection cycle starts with cell entry by clathrin-dependent and -independent mechanisms, followed by membrane fusion and virus uncoating, transcription and replication of the viral genome, translation and processing of viral proteins, virus assembly and genome packaging, and finally virus release (2). As the eight genomic segments of IAV encode only up to 17 polypeptides (3), some virus-encoded proteins, such as matrix protein 1 (M1), have different functions in the various steps of the IAV life cycle. In the mature virion, the M1 polymers form a shell at the inner surface of the envelope, where they interact with viral ribonucleoprotein (vRNP) complexes (4, 5) and the cytoplasmic tails of the viral glycoproteins hemagglutinin and neuraminidase, ensuring the structural integrity of influenza virions (6, 7). After virion internalization, IAVs are contained in endosomes, where the mildly acidic pH leads to M1 disassembly, a process that is supported by components of the aggresome processing machinery, such as histone deacetylase 6 (8). A nuclear localization sequence (NLS) in the M1 protein is important for the nuclear translocation of the freshly synthesized M1 (9). In the nucleus, M1 binds to the inducible phosphorylated nucleocapsid protein NP which, together with virion RNA and the heterotrimeric RNA-dependent RNA polymerase complex, forms active vRNPs. Nuclear export of the progeny vRNP requires not only the vRNP/M1 complex but also interaction with the viral nuclear export protein (NEP) at the host chromatin (10). NEP functions as an adaptor to enable nuclear export by the CRM1/exportin1-mediated pathway (11, 12) and caspase-dependent nuclear pore enlargement (13). The cytosolic vRNP travels toward the cell membrane along microtubules and in association with RAB11-positive recycling endosomes without being associated with M1 (14). The M1 protein is trapped in the vicinity of the cell membrane by interaction with the viral transmembrane protein M2 to support virus budding (15). The binding to lipid membranes together with a high local concentration of M1 can induce the formation of dimers and higher-order structures containing linear strands of M1 polymers (16). These linear M1 strands coil along the inside of the lipid bilayer and form filaments that are similar to those forming the endoskeleton of assembled virions and facilitate budding (17–19).

The intrinsic multifunctionality of M1 during the various steps of the IAV life cycle, together with its differential intracellular localization and multimerization state, requires its versatile regulation at multiple levels. These include differential and stage-specific protein-protein interactions, conformational flexibility, and the reversible attachment of different posttranslational modifications (PTMs) (20–24). Accordingly, the M1 protein can be modified by ubiquitination, SUMOylation, Neddylation, and phosphorylation (25–27). The occurrence of phosphorylation is typically temporally and spatially restricted by the controlled localization and the balanced activity of kinases and phosphatases. These enzymes can occur either in isolation or in protein assemblies of varying complexity, which enable additional levels of regulation and substrate recognition. One example for a highly organized multiprotein complex is provided by the evolutionarily conserved STRIPAK complex. This complex consists of a Striatin scaffold protein that tetramerizes to nucleate the assembly of a number of kinases (MST3, STK24, STK25, and MAP4K4), subunits of the PP2A phosphatase complex, and further regulatory proteins (28). This multifunctional signaling hub integrates diverse cellular signals into different pathways, such as Hippo signaling or autophagy (29, 30). Phosphorylations usually take place substoichiometrically to (i) ensure the sequential occurrence of biochemical processes and (ii) enable the occurrence of different subpopulations with distinct functions (31). Because the temporal and spatial dynamics of phosphorylation events are required for their regulatory function, both the absence of this modification as well as constitutive phosphorylation can lead to a loss of function.

Here, we have found that regulated M1 threonine 108 (T108) phosphorylation is required for IAV replication. Absent T108, phosphorylation prohibits its nuclear entry and M1-mediated nuclear vRNP export and leads to strongly increased self-association of M1. M1 T108 phosphorylation also controls association with host cell proteins, including members of the STRIPAK complex, which exerts a proviral function.

## RESULTS

### Regulated M1 T108 phosphorylation is essential for IAV replication.

**FIG 1:**
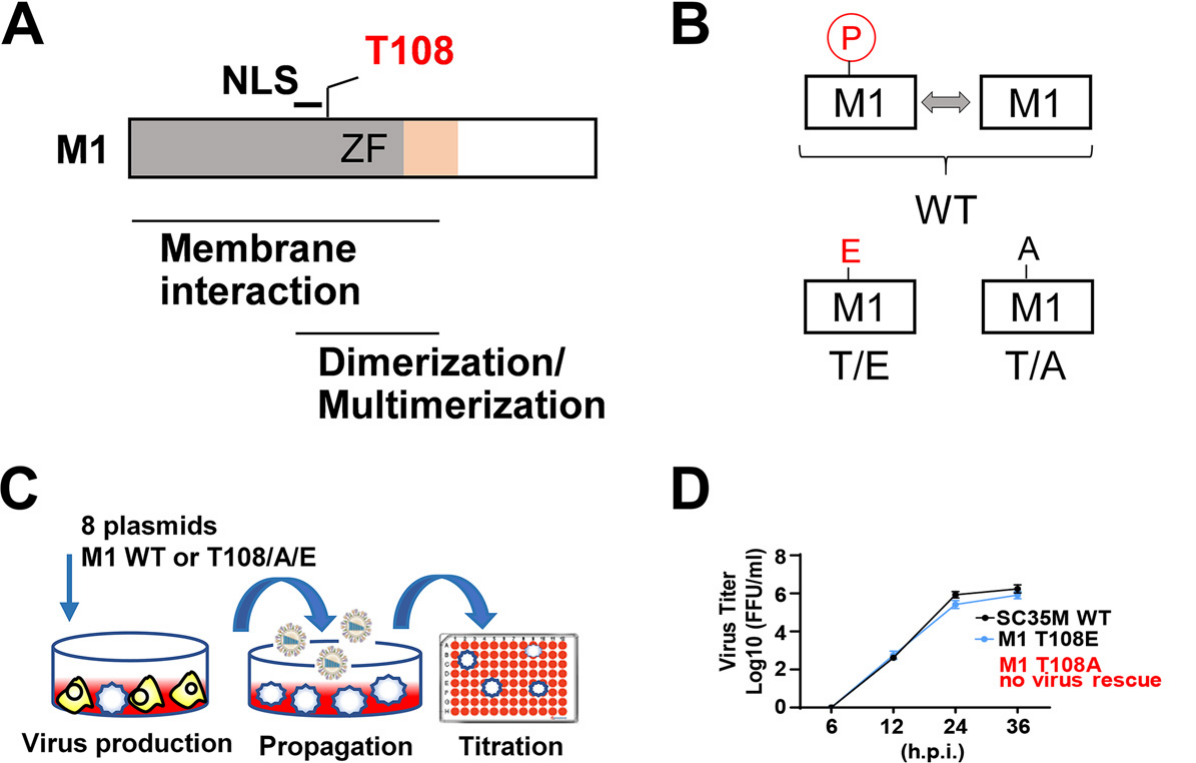
Phosphorylation at M1 T108 is required for viral rescue. (A) Schematic representation of the functional domains of IAV M1 protein; the NLS and the zinc finger (ZF) motif are shown. (B) Schematic display of the M1 T108 phospho mutants and the impact on the occurrence (T108A) or reversibility (T108E) of this phosphorylation. (C) Workflow visualizing creation and quantification of recombinant SC35M. (D) The recombinant virus was produced in 293T/MDCK-II cells and used to infect MLE-15 cells (MOI) = 0.001. Virus supernatant was harvested at the indicated time points, and viral titers were determined by foci assays. The virus titers are indicated, and bars show means ± standard deviations (SD) derived from three independent experiments performed in triplicate. n.s., not significant.

The M1 protein is phosphorylated at many positions including T108, as discovered by us for the mouse-adapted H7N7-type strain A/Seal/Massachusetts/1/80 (SC35M) and by others for the H1N1-type laboratory strain A/WSN/33 (WSN) (26, 32). This site is located within a region relevant for membrane association and multimerization that is adjacent to the NLS and highly conserved between different IAV strains from both avian and human species (Fig. 1A). To test a possible function of this M1 phosphorylation site, T108 was either changed to a phosphorylation-defective alanine (T/A) or a phospho-mimicking glutamic acid (T/E). This resulted in two mutants which lacked either the occurrence of this phosphorylation or the dynamic alternation between the unmodified and the phosphorylated state, as visualized in Fig. 1B. Reverse genetics using eight cotransfected plasmids encoding the viral proteins and RNAs was employed to create recombinant SC35M virus mutants using a coculture of 293T/MDCK-II cells, as schematically displayed in Fig. 1C. These experiments repeatedly failed to rescue viruses upon mutation of M1 T108A (Fig. 1D; see also Table S1 in the supplemental material), although control Western blotting experiments ensured the proper expression of the M1 protein and its mutants (Fig. S1). The M1 T108E phospho-mimicking SC35M mutant could be rescued (Fig. 1C, Table S1) and showed only a minor decrease in replication efficiency compared to wild-type (WT) SC35M in multicycle replication assays in MLE-15 cells (Fig. 1D). Inability to rescue M1 T108A-harboring viruses also occurred in Vero-E6 cells lacking type I interferon genes (Table S2) or when the M1 WT protein was coexpressed in *trans* (data not shown) (see Fig. S2A and B). Together, these experiments strongly suggested the functional relevance of regulated M1 T108 phosphorylation.

10.1128/mbio.03231-22.1 TABLE S1 Number and morphology of foci formed by SC35M WT and M1 T108 mutants. Supernatants from 293T/MDCK-II cells transfected with 8 plasmids were passaged on MDCK-II cells, followed by serial 1:10 dilutions. These were used to infect MDCK-II cells grown in 96-well plates, as schematically shown in Fig. 1C. One day later, cells were immunostained with monoclonal IAV anti-NP antibodies and horseradish peroxidase-conjugated secondary antibodies, followed by chromogenic development and image scanning using the BioSys Bioreader. Sheet 1 gives the number of foci from three independent experiments; sheet 2 displays the stained plates. Download Table S1, XLSX file, 2.7 MB. Copyright © 2023 Liu et al. 2023 Liu et al. https://creativecommons.org/licenses/by/4.0/ This content is distributed under the terms of the Creative Commons Attribution 4.0 International license.

10.1128/mbio.03231-22.2 TABLE S2 Number and morphology of foci formed by SC35M WT and M1 T108 mutants. Supernatants from 293T/Vero-E6 cells transfected with 8 plasmids were passaged on Vero-E6 cells, followed by serial 1:10 dilutions. These were used to infect Vero-E6 cells grown in 96-well plates, as schematically shown in Fig. 1C. One day later, cells were immunostained with monoclonal IAV anti-NP antibodies and horseradish peroxidase-conjugated secondary antibodies, followed by chromogenic development and image scanning using the BioSys Bioreader. Sheet 1 gives the number of foci from three independent experiments; sheet 2 displays the stained plates. Download Table S2, XLSX file, 2.3 MB. Copyright © 2023 Liu et al. 2023 Liu et al. https://creativecommons.org/licenses/by/4.0/ This content is distributed under the terms of the Creative Commons Attribution 4.0 International license.

10.1128/mbio.03231-22.5 FIG S1 Substitutions at M1 T108 did not affect the M1 protein expression levels. 293T cells were transfected to express the SC35M genome together with WT M1 or its phosphorylation-defective or phospho-mimicking versions. Extracts from cells lysed at 12 and 24 h.p.t. were further analyzed by immunoblotting for protein expression, as shown. Download FIG S1, PDF file, 0.1 MB. Copyright © 2023 Liu et al. 2023 Liu et al. https://creativecommons.org/licenses/by/4.0/ This content is distributed under the terms of the Creative Commons Attribution 4.0 International license.

10.1128/mbio.03231-22.6 FIG S2 Rescue experiments. (A) 293T/MDCK II cells were transfected to express the SC35M genome using bidirectional pHW2000 plasmids in an approach cotransfecting M1 WT together with M1 T108A. IAVs contained in supernatants were further characterized by titration, and virus progeny was isolated from individual plaques as shown schematically. Twelve plaques were purified, followed by amplification of the M1 gene using PCR and sequencing. (B) Results of the sequencing experiments are shown; the T108 codon is boxed. Download FIG S2, PDF file, 0.2 MB. Copyright © 2023 Liu et al. 2023 Liu et al. https://creativecommons.org/licenses/by/4.0/ This content is distributed under the terms of the Creative Commons Attribution 4.0 International license.

### Regulated M1 T108 phosphorylation is essential for its nuclear localization and efficient nuclear vRNP export.

**FIG 2:**
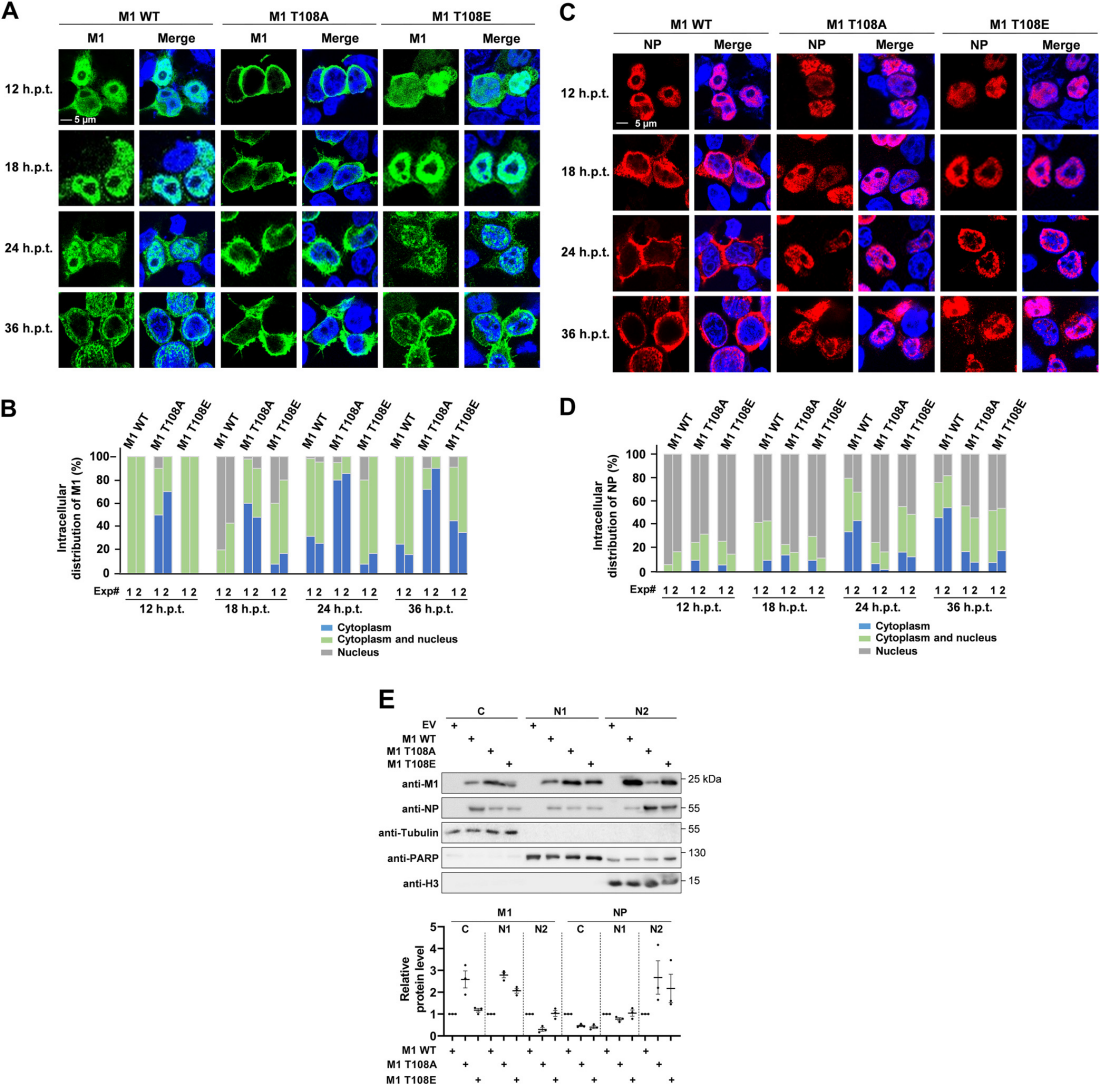
Phosphorylation of M1 T108 contributes to the nuclear import of M1 and nuclear export of vRNP. 293T cells were transfected using the 8-plasmid reverse genetic system for SC35M, and the intracellular localization of WT or mutant M1 was determined by immunofluorescence as shown. Nuclei were stained with Hoechst 33342 (blue); pictures are representatives of two independent experiments. (B) Each of the two independent experiments was analyzed separately for the intracellular distribution of M1 by analyzing 100 healthy interphase cells. (C and D) The experiment was performed as described for panels A and B, with the difference that the intracellular localization of NP was analyzed. (E) 293T cells were transfected under the same conditions as for immunofluorescence analysis or with empty vector (EV) as a control, followed by fractionation after 36 h.p.t. as shown. (Upper) Fractions were analyzed by immunoblotting for the distribution of the viral proteins, and the purity of the fractions was controlled by blotting for tubulin (C), PARP (N1), and histone H3 (N2). (Lower) Three independent experiments from panel E were used to quantify the M1 and NP protein amounts in the various fractions. The relative values were normalized to expression levels of the fraction markers; means ± standard errors of the means (SEM) are indicated.

As M1 T108 is located close to the NLS of the M1 protein (amino acids 101 to 105), a possible function of this phosphorylation for the regulation of M1 nuclear import and subsequent M1-dependent nuclear export of vRNPs was explored. In the absence of recombinant viruses expressing the M T108A mutant, 293T cells were transfected with 8 plasmids expressing all SC35M proteins including the WT M1 or its T108 phospho mutants. Indirect immunofluorescence revealed the localization of WT M1 in the cytosol and nucleus at 12 h posttransfection (h.p.t.) (Fig. 2A). Consistent with published data (33–35), the WT M1 was detected mainly in the nucleus at 18 h.p.t., before it localized to the cytosol and proximal to the cell membrane at later time points (Fig. 2A). In stark contrast, the M1 T108A mutant was largely excluded from the nucleus at all time points posttransfection. The dynamic time-dependent distribution of the M1 T108E mutant largely resembled that of the WT M1 protein, but with increased cytoplasmic localization 36 h.p.t., as seen by indirect immunofluorescence (Fig. 2A) and its quantitative analysis (Fig. 2B). Given the known contribution of M1 for nuclear export of the vRNP (11, 36), we scored the consequences of M1 phosphorylation for the localization of the NP protein, which represents the most abundant protein of vRNP and is widely used as a proxy for vRNP localization (37–39). Cells expressing the WT M1 protein showed the NP protein largely in the nucleus at 12 h.p.t. (Fig. 2C and D). Six hours later, the NP protein reached the nuclear periphery and was then transported to the cytosol at later time points (24 and 36 h.p.t.), as described previously (40). Cells expressing M1 T108A showed an enrichment of the NP protein in the nuclear periphery at 24 h.p.t., but its nuclear export at later time points was severely compromised (Fig. 2C and D). Cells expressing M1 T108E showed a reduced (but not absent) cytosolic localization of the NP protein, suggesting that regulated phosphorylation of M1 at T108 is critical for nuclear export of the vRNP. To substantiate these findings by an independent experimental approach, cell fractionation experiments were performed. Cells were transfected under the same conditions as for immunofluorescence analysis and fractionated into cytosolic (C), nucleoplasmic (N1), and chromatin (N2) extracts. These experiments confirmed the predominantly cytosolic localization of M1 T108A as well as NP retention in the chromatin fraction in the presence of M1 T108A and M1 T108E (Fig. 2E).

### M1 multimerization is restricted by T108 phosphorylation.

**FIG 3:**
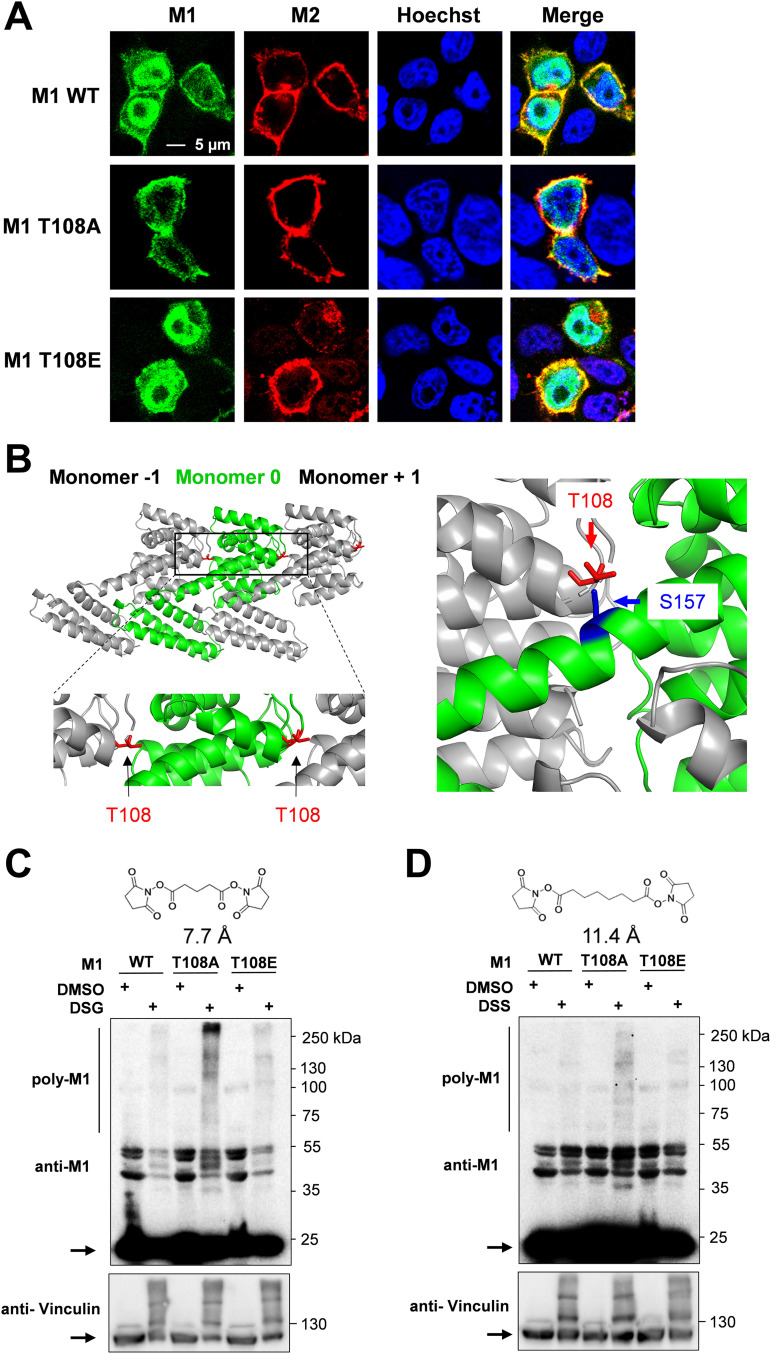
Absent M1 T108, phosphorylation promotes M1 polymerization. (A) 293T cells were transfected with an 8-plasmid reverse genetic system for SC35M expressing WT or mutant M1. Cells were fixed 24 h.p.t. and analyzed for localization of M1 (green) and M2 (red); nuclei were stained with Hoechst 33342 (blue), and areas of colocalization appear in yellow. Pictures are representative of three independent experiments. (B, left) The position of M1 T108 within the M1 polymer (PDB 1EA3) is shown in red; the boxed area is enlarged. (Right) T108 in monomer −1 is only 3. 8 Å away from S157, which is contained in helix 8 of the neighboring monomer 0. The structures were displayed using PyMOL. (C and D) 293T cells were transfected using the 8-plasmid reverse genetic system, and 1 day later cells were treated with the cross-linkers DSG (2 mM) (C) or DSS (2 mM) (D). Cell lysates were analyzed by Western blotting using specific antibodies. While monomeric proteins are indicated by arrows, the multimeric proteins appear as an upshifted smear. The positions of molecular weight markers are indicated.

We then determined the relevance of M1 phosphorylation for its localization to the membrane-proximal space, where it is found at later stages of the infection (41, 42). This was done using an indirect approach by costaining with the M2 protein, which assists M1 transport to the plasma membrane region via a piggyback mechanism (15). Human 293T cells were transfected with 8 plasmids expressing all SC35M proteins including the WT or phospho mutant M1 proteins, and the intracellular localization of the M1 and M2 proteins was detected by indirect immunofluorescence. The M1 protein and its mutants colocalized with the M2 protein in the membrane-proximal space, but the majority of M1 WT and M1 T108E localized in the nucleus (Fig. 3A).

Association of M1 with the plasma membrane triggers its oligomerization as a consequence of an increased affinity to other monomers, ultimately providing structural integrity and stability to the virion (43–45). The position of M1 T108 within the protein suggested a possible role in the regulation of M1-M1 interactions (Fig. 3B) (18, 19). In order to investigate the possible impact of M1 T108 phosphorylation on M1 polymerization, cross-linking experiments were performed. 293T cells were transfected to express the SC35M genome together with WT M1 or its phosphorylation-defective or phospho-mimicking versions. Proteins in close proximity were cross-linked using two membrane-permeable, bifunctional lysine-to-lysine cross-linkers which differed in lengths of their spacer arms, namely, disuccinimidyl glutarate (DSG) or disuccinimidyl suberate (DSS). After cross-linking, M1 multimerization was revealed by Western blotting where cross-linked protein species appeared as multiple upshifted bands. The M1 T108A protein showed strongly increased self-association after cross-linking with DSG (Fig. 3C) and DSS (Fig. 3D), indicating that dynamic phosphorylations restricted the ability of M1 for multimerization.

### Identification of the M1 interactome reveals its association with the STRIPAK complex.

**FIG 4:**
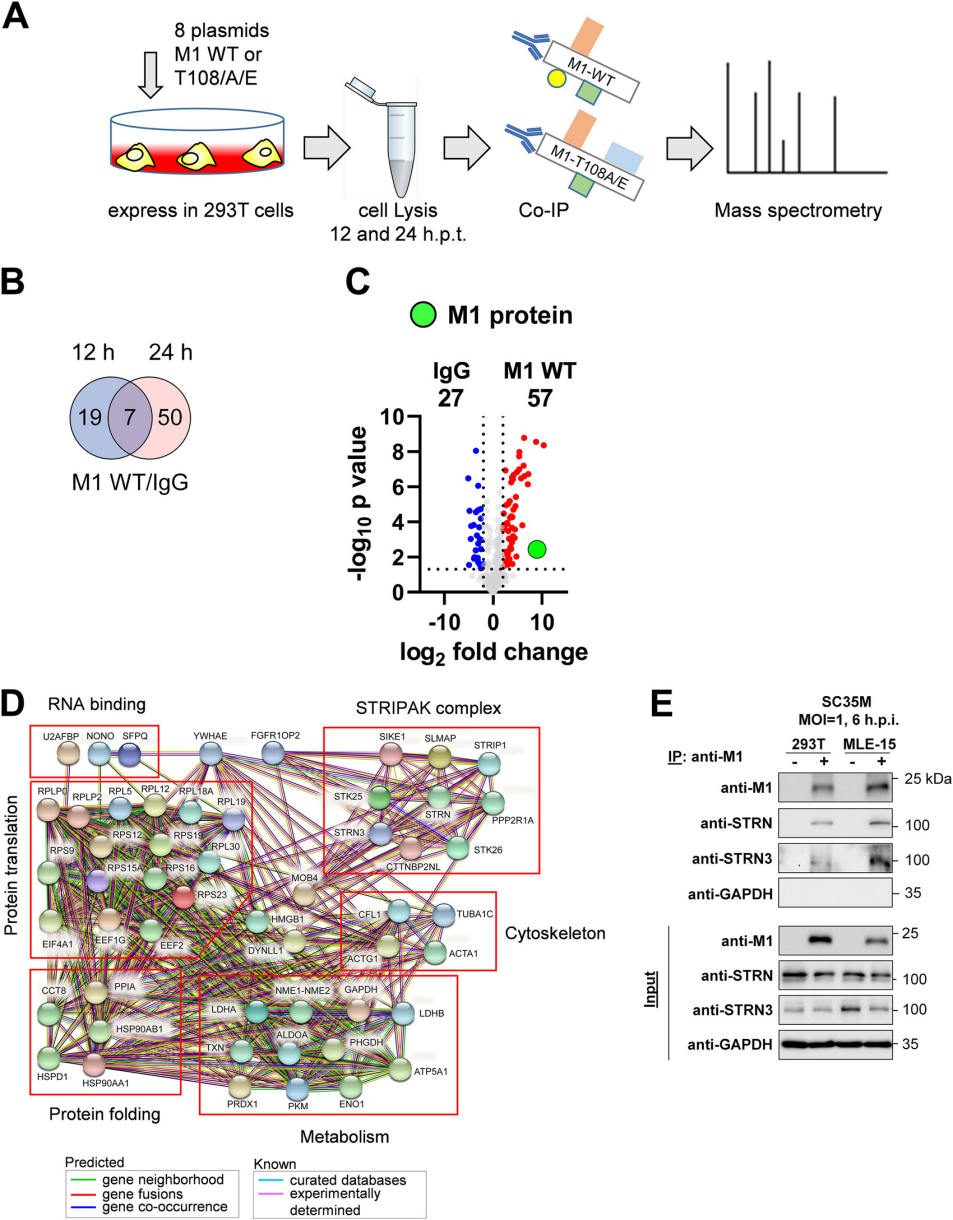
Identification of the M1 interactome. (A) Schematic display of the experimental setup used to identify proteins binding to M1 WT or its T108 phospho mutants. 293T cells expressing 8 plasmids encoding SC35M together with M1 WT, M1 T108A, or M1 T108E were lysed. An aliquot of the lysates was used for immunoblotting to ensure proper and comparable expression of M1, while the remaining material was subjected to co-IP using anti-M1 antibodies or a nonspecific control rabbit IgG. The immunoprecipitates were subsequently subjected to mass spectrometric analysis; data were acquired from three biological replicates. (B) Proteins interacting with M1 at 12 or 24 h.p.t. were identified by comparison to the IgG controls, and specific interaction with M1 was defined to occur with an LFC of ≥1 in a statistically significant manner (*P ≤ *0.05). These analyses yielded M1 interactors for both time points; a comparison is shown in the Venn diagram. (C) The M1 interactomes of the cells expressing M1 WT for 24 h.p.t. were subjected to pairwise comparisons with the IgG control by using Volcano plots. Specific M1 interactors (LFC of ≥1; *P ≤ *0.05) are shown in red, and M1 is highlighted in green. (D) The STRING database (version 11.5) was used to reveal known and predicted interactions between the M1 interactors; the proteins were grouped according to their biological functions, as shown. (E) 293T cells or MLE-15 cells infected with SC35M (MOI = 1) were harvested at 6 hpi, and cell lysates were prepared. One fraction of the lysate was used as an input control, while the remaining material was used for co-IP experiments using anti-M1 antibodies, as shown. The indicated proteins were detected by Western blotting analysis with specific antibodies.

To investigate a potential effect of regulated M1 T108 phosphorylation on protein-protein interactions by an unbiased approach, we aimed to identify the M1 interactome by mass spectrometry, as illustrated in Fig. 4A. 293T cells were transfected with 8 plasmids to express the SC35M genome together with the WT M1 or its T108 phospho mutants. Cell lysates were prepared after 12 or 24 h, and one aliquot of the extracts was used for control Western blot assays to ensure comparable M1 expression (Fig. S3), while the remaining material was used for further analysis by coimmunoprecipitation (co-IP) and mass spectrometry. The results from three independent biological experiments are given in Table S3 and showed that the number of specific M1 interactors was much larger after 24 h of protein expression compared to the number of interactors found after 12 h.p.t. (Fig. 4B). Thus, we focused for further analysis on interactors binding significantly (*P ≤ *0.05) to M1 WT (fold change of ≥2-fold, corresponding to a log_2_ fold change [LFC] of ≥1) after 24 h of protein expression (Fig. 4C). These 57 significant interactors could be assigned to different functional categories, with the largest group consisting of proteins that mediate protein translation and folding and further groups involved in regulation of metabolism and components of the cytoskeleton (Fig. 4D), all representing functional categories that are known to be affected by IAV infection or proteins binding to IAV-encoded polypeptides (22, 46–49). Some of the M1 interactors identified here are known to contribute to influenza virus replication, including RPS3 (50), PHGDH (51), and RPS26 (52). The analysis of the M1 interactome also detected 10 components of the STRIPAK complex, which has been implicated in many physiological processes, such as neuronal and embryonic development, proliferation, and cell survival, while its role in virus replication is not known (53, 54). Together, these data show the interaction of M1 with a number of host cell proteins, including members of the regulatory STRIPAK complex. To investigate the interaction of the STRIPAK complex with M1, we focused on the Striatin family members Striatin (STRN) and Striatin-3 (STRN3), which were identified in the interactome analysis and act as central platforms to organize the assembly of entire STRIPAK complex (28). Binding of STRN and STRN3 to the M1 protein was also observed by a complementary experimental approach using co-IP after infection of human 293T cells or murine MLE-15 cells with SC35M viruses (Fig. 4E).

10.1128/mbio.03231-22.3 TABLE S3 Summary of the co-IP MS experiments. 293T cells expressing 8 plasmids encoding SC35M together with M1 WT, M1 T108A, or M1 T108E were lysed and subjected to co-IP using anti-M1 antibodies or a nonspecific control rabbit IgG. The immunoprecipitates were subsequently subjected to mass spectrometric analysis. Data were acquired from three biological replicates. For details see text. Download Table S3, XLSX file, 2.3 MB. Copyright © 2023 Liu et al. 2023 Liu et al. https://creativecommons.org/licenses/by/4.0/ This content is distributed under the terms of the Creative Commons Attribution 4.0 International license.

10.1128/mbio.03231-22.7 FIG S3 Quality check of M1 expression before analysis by mass spectrometry. 293T cells expressing 8 plasmids encoding SC35M together with M1 WT, M1 T108A, or M1 T108E were lysed. While 90% of the material was used for co-IP and mass spectrometry as described in the legend for Fig. 4, the remaining material analyzed here was used for immunoblotting to ensure comparable and proper expression of M1. Results from all three biological replicates are shown. Download FIG S3, PDF file, 0.1 MB. Copyright © 2023 Liu et al. 2023 Liu et al. https://creativecommons.org/licenses/by/4.0/ This content is distributed under the terms of the Creative Commons Attribution 4.0 International license.

### Dynamic regulation of protein-protein interactions and STRIPAK binding by M1 T108 phosphorylation.

**FIG 5:**
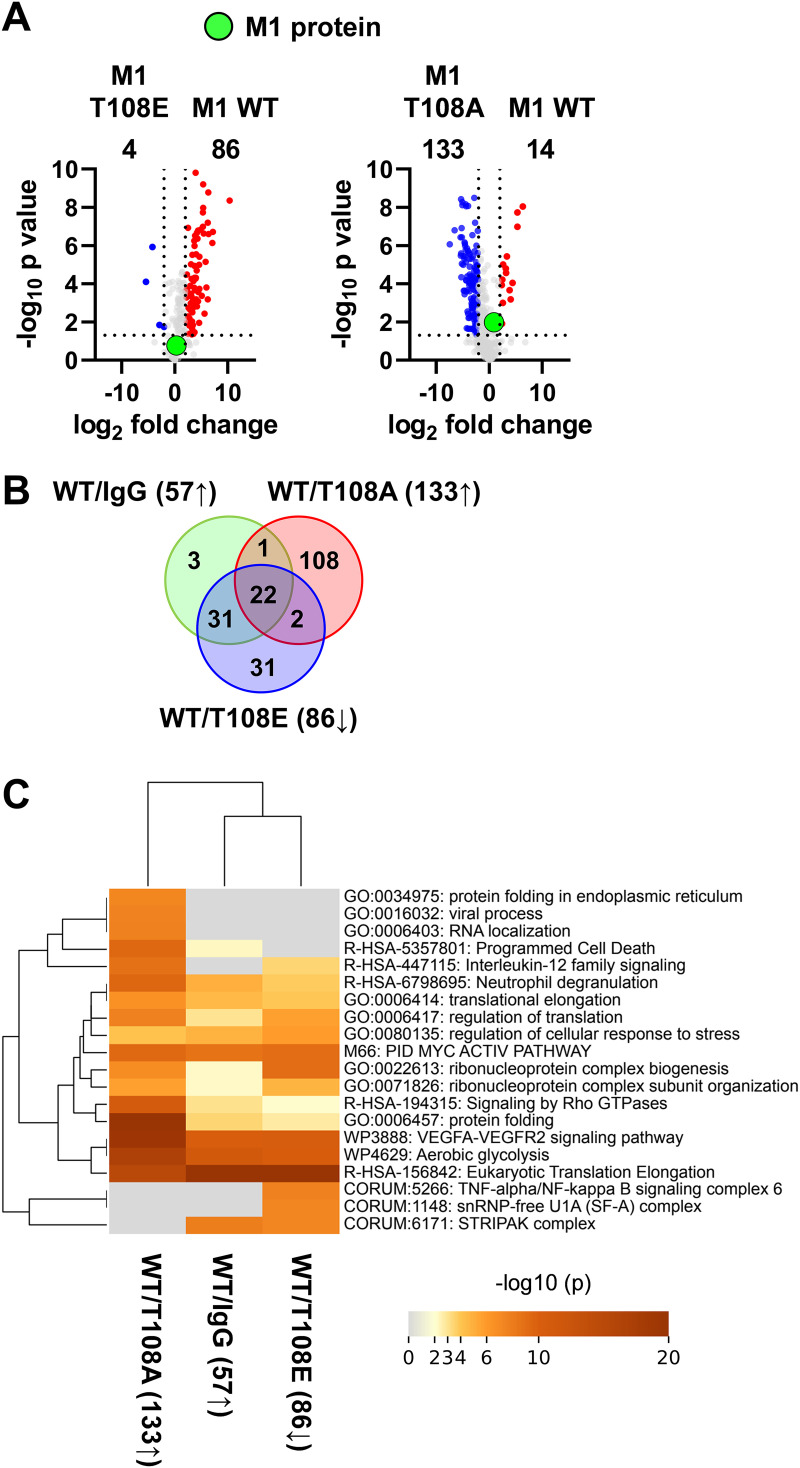
M1 T108 phosphorylation impairs the interaction of M1 with other proteins. (A) Volcano plots depicting pairwise comparisons of the interactomes from cells expressing M1 WT with cells expressing M1 T108A or M1 T108E (LFC, ≥1; *P ≤ *0.05). (B) Venn diagrams visualizing the overlap of protein binding to M1 WT and its phospho mutants. (C) Overrepresentation analysis for enriched M1 interactors. Numbers in brackets correspond to the protein sets shown in panel B.

**FIG 6:**
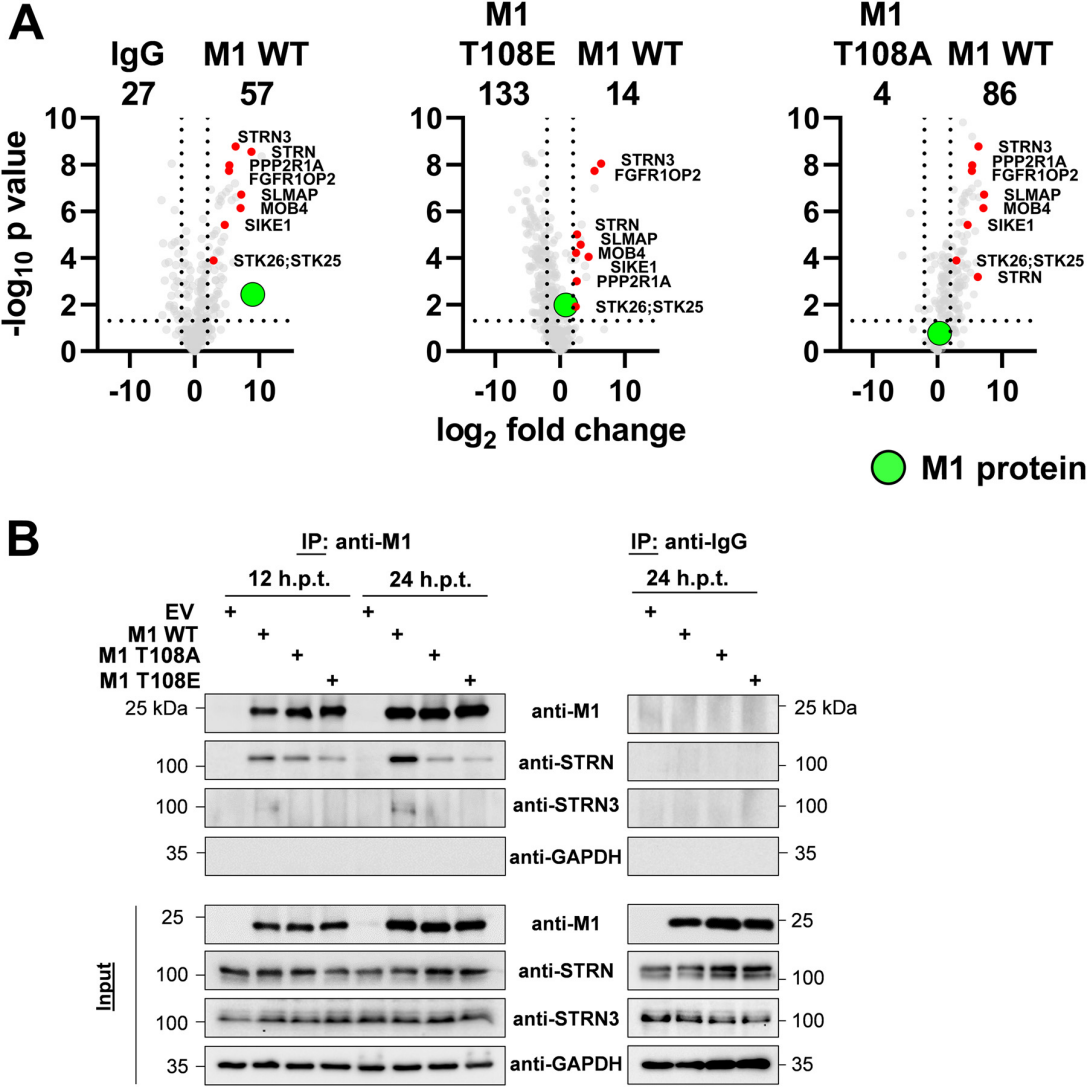
Preferential binding of STRN and STRN3 to M1 WT. (A) Pairwise comparison of the interactomes from cells expressing M1 WT with cells expressing M1 T108A or M1 T108E (LFC, ≥1; *P ≤ *0.05). Members of the STRIPAK complex are highlighted in red, and M1 is shown in green. (B) 293T cells were transfected with an 8-plasmid reverse genetic system for SC35M expressing WT or mutant M1 as shown. Lysates were prepared after 12 or 24 h, and one part of the lysates was used for the input control. The remaining material was subjected to co-IP using M1 antibodies or IgG controls, as shown. The expressed and precipitated proteins were detected by Western blotting analysis with specific antibodies; a representative experiment is shown.

We then compared the impact of absent or constitutive T108 phosphorylation on the M1 interactome. A comparison of the interactome between M1 WT and M1 T108E showed that the phospho-mimicking version of this viral protein had largely lost its ability for interactor binding. In contrast, the M1 T108A mutant had an increased capacity to interact with cellular proteins (Fig. 5A). These interactors comprised most of the proteins binding to M1 WT, but in addition the M1 T108A protein bound to further 110 proteins which did not belong to the group of high-affinity WT M1 interactors (Fig. 5B). Mapping of the proteins interacting with M1 WT to GO/KEGG pathways using Metascape software revealed that these proteins mediate functions such as protein synthesis and metabolic regulation. Furthermore, pathway mapping showed that proteins additionally associating with M1 T108A are involved in regulation of the interleukin 12 pathway and protein folding (Fig. 5C). The small number of M1 interactors found at 12 h.p.t. did not allow statistically robust conclusions to be drawn about the effects of phosphorylation on protein-protein interactions. The analysis of protein-protein interaction data by volcano plots indicated that all members of the STRIPAK complex showed preferential interaction with the M1 WT protein. Significantly reduced binding of individual STRIPAK components was seen for the M1 mutant defective in the dynamics of this modification (T108E), while slightly diminished interactions were seen for association with M1 T108A (Fig. 6A). The preferential interaction of M1 WT with STRN and STRN3 was also recapitulated by co-IP experiments after transient expression of the interaction partners (Fig. 6B).

### IAV-mediated relocalization of STRN and STRN3.

**FIG 7:**
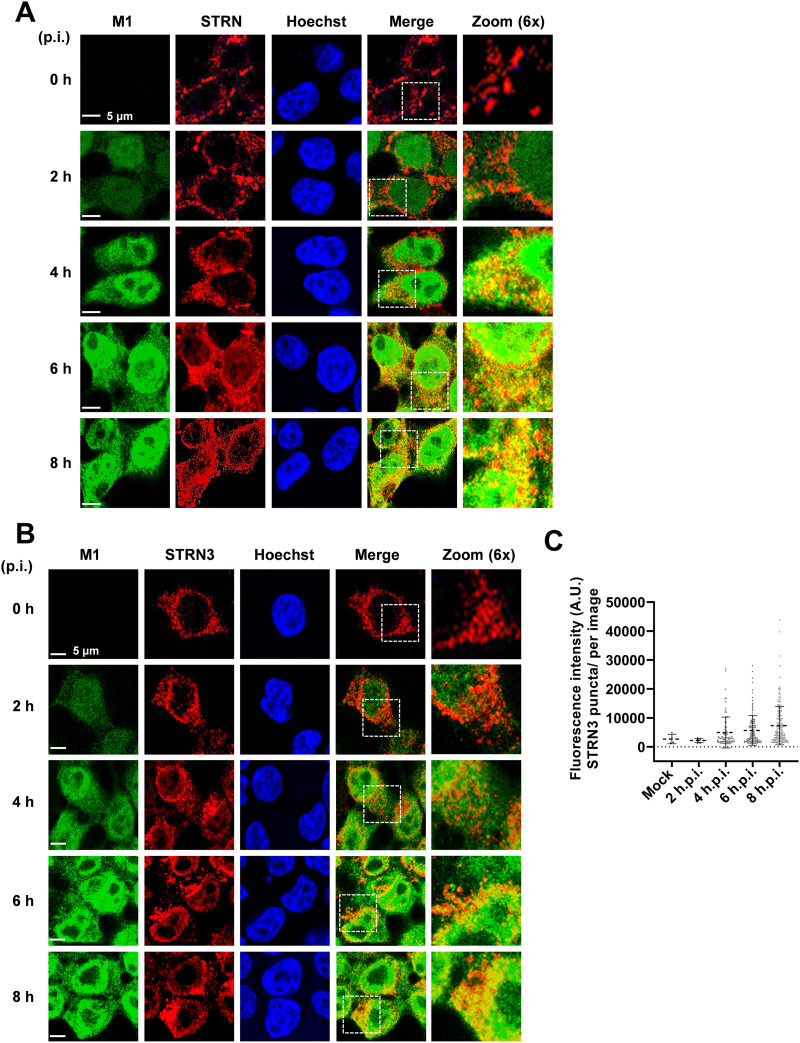
IAV-mediated relocalization of STRN and STRN3. (A) 293T cells were infected with SC35M (MOI = 2) for various periods as shown. STRN and the viral protein M1 were detected by indirect immunofluorescence, and nuclei were revealed by staining of nuclear DNA with Hoechst dye. Representative confocal images from two independent experiments are shown; yellow areas indicate colocalization, and the boxed areas are displayed in 6× magnification. Representative confocal images from two independent experiments show intracellular colocalization between STRN and the viral M1 protein. (B) The same experiment was performed as described for panel A, with the exception that M1 was costained with STRN3. (C) Quantification of the number of STRN3 puncta per image. Data are means ± SD from one representative experiment where at least 150 cells per condition were quantified. A.U., arbitrary units.

To examine a possible colocalization between M1 and both striatins, 293T cells were infected with SC35M, and the intracellular localization of proteins was revealed by indirect immunofluorescence. Consistent with published studies, STRN was mainly detected at the cell membrane of uninfected cells (55, 56). IAV infection led to the drastic redistribution of this adapter protein to cytosolic and perinuclear structures, where it partially colocalized with M1 (Fig. 7A). A dynamic and IAV-induced relocalization also occurred for STRN3. While it occurred as a cytosolic protein in uninfected cells, as described previously (57), IAV infection led to the formation of STRN3 clusters in the perinuclear region starting at 4 hpi (Fig. 7B). As infection progressed, these domains continuously increased in size and density, forming superclusters that were asymmetrically enriched on one side of the perinuclear space, as displayed in Fig. 5C and quantified in Fig. 5D. Colocalization between STRN3 and M1 was most prominent in the perinuclear space as well as in these superclusters. Because these experiments showed an IAV-mediated relocalization of STRN and STRN3, it was interesting to investigate whether, *vice versa*, these adapter proteins had an impact on IAV replication.

### STRN- and STRN3-mediated support of IAV infection.

**FIG 8:**
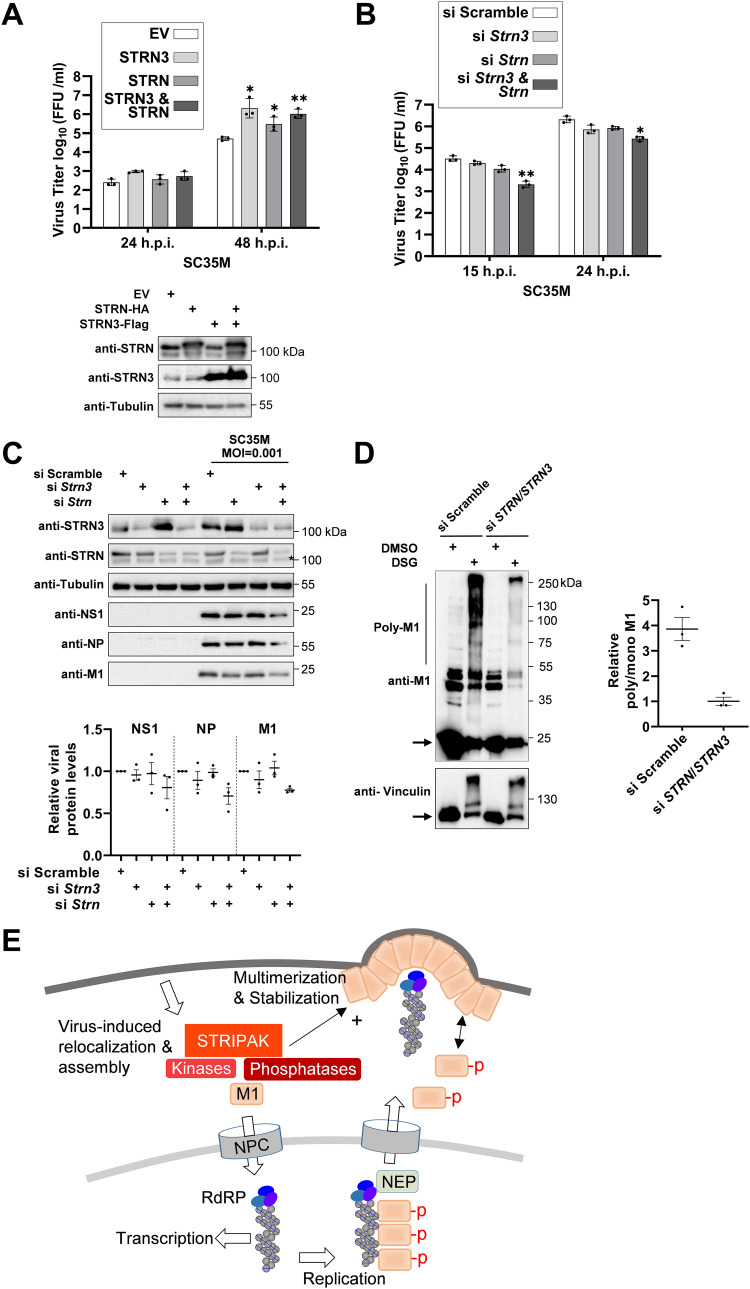
STRN- and STRN3-mediated support of IAV replication. (A) 293T cells transfected to express STRN or STRN3 or empty vector (EV) as a control. (Upper) One fraction of the cells was infected with SC35M (MOI = 0.001), and viral titers were determined at the indicated time points. Bars indicate means ± SD obtained from three independent experiments performed in triplicates. *, *P* ≤ 0.05; **, *P* ≤ 0.01. (Lower) Another fraction of the cells was analyzed by immunoblotting for expression of STRN or STRN3. (B) MLE-15 cells were seeded and transfected 1 and 3 days later with siRNAs targeting mRNAs encoding STRN or STRN3 or an adequate scrambled control as shown. (Left) After 3 days cells, remained untreated or were infected with SC35M (MOI = 0.001) as shown. The cells were infected with SC35M (MOI = 0.001) and viral titers of cell culture supernatants were determined at the indicated time points. Bars indicate means ± SD obtained from three independent experiments performed in triplicates. *, *P* ≤ 0.05; **, *P* ≤ 0.01; unpaired *t* test. (C) The knockdown cells generated in panel B were tested by Western blotting for adequate knockdown and expression of viral proteins as shown; the position of a nonspecific band is highlighted by an asterisk. The lower part shows quantification of three independent experiments; expression in the controls was set to 1, and means ± SEM are indicated. (D) The expression of STRN and STRN3 in 293T cells was reduced by siRNA-mediated knockdown, and cells were infected with SC35M (MOI = 3). (Left) At 6 hpi, cells were treated with DSG and lysates were analyzed for M1 multimerization by Western blotting, similar to Fig. 3C and D. (Right) Three independent experiments were used to calculate the intensity ratios of polymeric to monomeric M1 proteins. Means ± SEM are indicated. (E) Schematic summary of possible functions of M1 T108 phosphorylation investigated in this study. M1 T108 phosphorylation is required for the complete nuclear import of M1 and nuclear export of vRNP, and it affects the ability of M1 for homopolymerization, a process that is also controlled by the STRIPAK complex.

Overexpression of STRN or STRN3 led to increased SC35M titers, while combined expression of both proteins did not result in a further increase of virus replication (Fig. 8A). In a complementary approach, the role of both adapter proteins was investigated in MLE-15 cells or 293T cells, where both proteins were knocked down with small interfering RNAs (siRNAs). Depletion of STRN or STRN3 alone had no effect on SC35M replication, while the combined knockdown of STRN together with STRN3 resulted in reduced viral titers, confirming the proviral function of STRN and STRN3 (Fig. 8B, Fig. S4). The control Western blot experiments ensuring successful knockdown showed that downregulation of STRN led to increased STRN3 levels and, vice versa, downregulation of STRN3 resulted in increased STRN amounts (Fig. 8C). This behavior suggests a mutual compensatory mechanism for both adapter proteins, a feature that is frequently observed in multiprotein complexes (58, 59). These blots and their quantitative analysis also showed that the double knockdown led to a slight but significant decrease in the levels of viral proteins (M1, NS1, and NP) (Fig. 8C). The relevance of STRN and STRN3 for IAV replication was also found in cells infected with the human IAV subtype A/Hamburg/4/09 (H1N1_pdm09_), which caused the 2009 pandemic (60). Also in this model, simultaneous knockdown of STRN and STRN3 caused reduced virus titers and slightly decreased expression of viral proteins (Fig. S5), indicating that the virus-supportive function of the STRIPAK complex is not restricted to one IAV strain. It was then interesting to investigate the molecular mechanisms underlying STRN- and STRN3-mediated support of IAV replication. STRN and STRN3 knockdown resulted in severely compromised M1 multimerization in SC35M-infected cells (Fig. 8D).

10.1128/mbio.03231-22.8 FIG S4 Effect of STRN and STRN3 on SC35M replication in 293T cells. Expression of STRN and STRN3 was reduced upon knockdown with specific siRNAs. Cells were infected with SC35M virus (MOI = 0.001), and the viral titers were determined at the indicated time points. Bars indicate means ± SD obtained from three independent experiments performed in triplicate. **, *P* ≤ 0.01. Download FIG S4, PDF file, 0.1 MB. Copyright © 2023 Liu et al. 2023 Liu et al. https://creativecommons.org/licenses/by/4.0/ This content is distributed under the terms of the Creative Commons Attribution 4.0 International license.

10.1128/mbio.03231-22.9 FIG S5 Effect of the Striatin complex on replication of H1N1pdm09. MLE-15 cells were seeded and transfected 1 and 3 days later with siRNAs targeting mRNAs encoding *Strn/Strn3* or an adequate scrambled control as shown. (Left) One fraction of the cells was infected with SC35M (MOI = 0.001), and viral titers of cell culture supernatants were determined at the indicated time points. Bars indicate means ± SD obtained from three independent experiments performed in triplicates. *, *P* ≤ 0.05; **, *P* ≤ 0.01 by unpaired *t* test. (Right) The remaining fraction of the cells was lysed and tested by Western blotting for adequate knockdown and expression of viral proteins as shown. The position of a nonspecific band is highlighted by an asterisk. The lower part shows a quantification of protein expression from three independent experiments. Expression in the controls was set to 1. The means ± SEM are indicated. Download FIG S5, PDF file, 0.2 MB. Copyright © 2023 Liu et al. 2023 Liu et al. https://creativecommons.org/licenses/by/4.0/ This content is distributed under the terms of the Creative Commons Attribution 4.0 International license.

## DISCUSSION

The M1 protein functions as a multifunctional “Swiss army knife,” as it exerts different functions including the maintenance of virion integrity, inhibition of viral transcription, assistance in vRNP nuclear export, and endowment of virus assembly and budding (11, 42, 61). Accordingly, this viral protein is found in different subcellular localizations and must interact with distinct cellular and viral proteins, depending on the localization and stage in the infection cycle. This functional versatility is reflected at the biochemical level by its intrinsic structural flexibility (20, 61), its ability for reversible assembly and disassembly, and by the occurrence of regulated PTMs. These modifications affect many of the different M1 functions, including virus replication (Neddylation) and vRNP nuclear export (ubiquitination) (27, 62). Phosphorylation has been described for a number of amino acids in M1 (26, 32, 33, 63), but only a few phosphorylation sites have been characterized in depth. For example, M1 phosphorylation at Y132 controls nuclear import of M1 (33). This phosphorylation is likely mediated by Janus kinases late upon infection and probably induces structural changes that facilitate M1 lipid raft localization and efficient vRNP incorporation into budding virions (23). Our study revealed that the control of M1 multimerization involves phosphorylation at T108, probably by disturbing the interaction of T108 in monomer 0 with S157 contained in helix 8 of the interacting +1 monomer. M1 multimerization can also be restricted by known phosphorylation sites in the N-terminal region (S2/T5 and T9/Y10) (45), but experimental proof for their potential role in M1 multimerization has not yet been obtained. We propose that phosphorylation at T108 and also other residues allows for signal-guided, regulated, and reversible control of the M1 multimerization state. Such a mechanism can contribute to M1 shell release from the endosome surface early during infection, inhibit premature multimerization that would prohibit nuclear import, and allow proper timing of multimerization at the cell membrane. This model would explain why defects in the occurrence (M1 T108A) or reversibility (M1 T108E) of this phosphorylation can result in functional deficiencies. The increased multimerization of the M1 T108A mutant would not only prohibit its nuclear import but potentially also explain why the M1 T108A mutant displays an increased spectrum of interactors. It will therefore be an important task in the future to define the M1 interactome in a timely and spatially resolved fashion and in dependence of its oligomerization state. These parameters are codefining criteria for the M1 interactome and might also explain why previous interactome studies for M1 delivered remarkably heterogeneous results (64). A deeper understanding of M1-interacting proteins will therefore require experimental approaches allowing determinations of protein-protein interactions within intact cells, such as proximity ligation assays (65).

The association of M1 with components of the STRIPAK complex discovered in this study occurs outside of the nucleus in distinct membrane-proximal and cytosolic regions, with STRN3 colocalizing with M1 in asymmetric but undefined superclusters. Such an asymmetric distribution has also been described for RAB11 endosomes (66), but members of the STRIPAK complex have been found in a remarkably large number of different cellular compartments, including the Golgi apparatus, nuclear envelope, autophagosomes, mitochondria, and cell membrane (53). This distribution has led to the suggestion of STRIPAK functioning as a bridging complex to connect these different organelles (67). The molecular mechanisms underlying IAV-mediated redistribution of intracellular STRN and STRN3 localization are not clear and might depend on the massive reorganization of the actin and tubulin cytoskeleton that occurs during IAV infection (68). Of note, STRIPAK subcomplexes can govern actin assembly (53), and M1 has also been found as an interaction partner of actin (49). The interaction of M1 with the STRIPAK complex might enable the transmission of M1-derived signaling events to STRIPAK-controlled networks, which need to be explored in the future. The decrease of virus titers in the absence of STRIPAK is due neither to changes in IAV polymerase activity nor to changes in vRNP nuclear export (Fig. S6A and B). The contribution of the STRIATIN complex for IAV replication instead involves the support of M1 multimerization, probably by assisting in the dephosphorylation of M1 via its associated PP2A phosphatases, as shown schematically in Fig. 8E. Phosphorylation of M1 T108 by STRIPAK-associated kinases is unlikely, as otherwise the STRN and STRN3 knockdown would result in absent M1 T108 phosphorylation, which is incompatible with viral replication. The sequence surrounding the M1 T108 LKREI(T*)FHGAKE phosphorylation site is more likely to be phosphorylated by kinases belonging to the AGC kinase group, such as the PKC family members PDK1 and PKN1, which preferentially phosphorylate motifs containing a K/R at position −3 and an F at +1 (69). The identification of relevant kinases is thus an important future task to allow pharmacological targeting of individual steps during IAV infection, which is an important future goal to enable new strategies for antiviral intervention.

10.1128/mbio.03231-22.10 FIG S6 Effect of STRN and STRN3 on polymerase activity and localization of viral proteins. (A) Expression of STRN and STRN3 was reduced upon knockdown with specific siRNAs in 293T cells. (Left) After knockdown, the cells were transfected to express a Pol I promoter-driven viral RNA-like transcript encoding the reporter gene together with pCAGGS plasmids, allowing the expression of mRNAs encoding the indicated viral proteins. One day later, cells were harvested and activities from firefly luciferase (generated by vRNP polymerase activity) and *Renilla* luciferase (used for normalization) were determined. Activity of the WT vRNP was set to 100%. Error bars represent SEM from three independent experiments. n.s., not significant. (Right) A portion of the cells was analyzed by immunoblotting for successful knockdown as shown. (B) Another fraction of the siRNA-treated cells was infected by SC35M (MOI = 3), and intracellular localization of the indicated proteins was determined by indirect immunofluorescence as shown. Nuclei were stained with Hoechst 33342 (blue). Pictures are representatives of two independent experiments. Download FIG S6, PDF file, 0.4 MB. Copyright © 2023 Liu et al. 2023 Liu et al. https://creativecommons.org/licenses/by/4.0/ This content is distributed under the terms of the Creative Commons Attribution 4.0 International license.

## MATERIALS AND METHODS

### Antibodies, primers, and plasmids.

The antibodies, primers, and plasmids used in this study are provided in Table S4 in the supplemental material.

10.1128/mbio.03231-22.4 TABLE S4 Antibodies, primers, and plasmids used in this study. Download Table S4, DOCX file, 0.02 MB. Copyright © 2023 Liu et al. 2023 Liu et al. https://creativecommons.org/licenses/by/4.0/ This content is distributed under the terms of the Creative Commons Attribution 4.0 International license.

### Cell culture and siRNA transfection.

Human embryonic kidney 293 T cells, Madin-Darby and canine kidney cells (MDCK-II), Vero-E6 cells, and murine MLE-15 lung epithelial cells were grown in Dulbecco’s modified Eagle medium (DMEM) supplemented with 10% (vol/vol) fetal calf serum, 100 U/mL penicillin, and 100 μg/mL streptomycin. Cells were grown in an incubator at 37°C in a humidified atmosphere containing 5% (vol/vol) CO_2_. Predesigned siRNAs targeting human or murine STRN and STRN3 were transfected into MLE-15 cells using Lipofectamine 3000 (Invitrogen) diluted in Opti-MEM (Life Technologies) according to the manufacturer's protocols. The medium was replaced with fresh growth medium 24 h.p.t. cells were then transfected for a second time at 48 h following the first transfection, using the same protocol. The cells were further incubated at 37°C for 48 h prior to either infection or lysis and Western blotting.

### Generation of recombinant IAVs.

The WT SC35M virus and its M1 mutants were generated by using a set of eight plasmids based on the bidirectional pHW2000 plasmid reverse genetics system, described elsewhere (32). Plasmids encoding M1 mutated at position 108 were generated by site-directed mutagenesis. A coculture of 293T and MDCK-II cells (ratio 3:1) was grown to 70% confluence and transfected with 1 μg DNA of each plasmid encoding the eight viral segments by using Opti-MEM (Life Technologies) and Lipofectamine 2000 (Invitrogen). The rescue of the SC35M M1 T108A mutant was also attempted upon transfection with nine plasmids coexpressing M1 T108A (1 μg) with WT M1 (0.5 μg) in 293T cells. The medium was removed 6 h.p.t., and fresh DMEM supplemented with 0.2% (wt/vol) bovine serum albumin (BSA), 100 U/mL penicillin, and 100 μg/mL streptomycin (Life Technologies) was added. Cells were monitored for occurrence of the cytopathic effect every 24 h. The cell culture supernatant was harvested 48 h.p.t., and cell debris was removed by centrifugation. A 500-μL aliquot of each supernatant was used to inoculate MDCK-II or Vero-E6 cells, which were then incubated for 48 to 72 h. The viruses rescued from these cells were titrated by foci assays in MDCK-II or Vero-E6 cells in 96-well plates and stored at −80°C until further use, as described elsewhere (70).

### Plaque purification and M1 sequencing.

Virus-containing culture supernatants from 293T cells transfected with nine plasmids coexpressing M1 WT and the M1 T108A mutant were analyzed by foci assays. Monolayers of MDCK-II cells in 6-well dishes were washed with phosphate-buffered saline (PBS) and infected with the virus-containing supernatant and various 10-fold serial dilutions in the infection medium (DMEM supplemented with 0.2% [wt/vol] BSA and penicillin-streptomycin) and incubated at 37°C for 1 h. The wells were aspirated to remove residual viral inoculum, and the MDCK-II cells were overlaid with 2 mL 1× agarose overlay mixture (minimal essential medium supplemented with penicillin-streptomycin, 0.4% [wt/vol] BSA, 1% [wt/vol] agarose). After the agar overlay solidified, the plates were incubated at 37°C with 5% CO_2_ upside down for 2 days. The plaques were harvested to infect MDCK-II cells for propagation of viruses from the purified plaques. Viral RNA was extracted from 140-μL aliquots of supernatants of virus-infected cells using a QIAamp viral minikit (Qiagen, Germany) according to the manufacturer’s protocol. The extracted viral RNA was reverse transcribed and amplified by PCR in a single reaction using a SuperScript IV One-Step reverse transcription-PCR (RT-PCR) system (Invitrogen). Briefly, 7.5 μL of extracted viral RNA was mixed with 25 μL 2× Platinum SuperFi RT-PCR master mix, 0.5 μL of enzyme mix, and M1-specific primers (0,4 μM each). The total volume was adjusted to 50 μL using nuclease-free water and subjected to cDNA synthesis at 55°C for 10 min, followed by predenaturation (98°C for 2 min) and PCR amplification. The different PCR products containing the SC35M M1 gene were purified from agarose gels and sequenced.

### Virus propagation, infection, and titer determination.

293T and MLE-15 cells were infected with IAV SC35M (H7N7) viruses or A/Hamburg/4/09 (H1N1) in PBS++/BSA (PBS containing 0.2% [wt/vol] BSA, 1 mM MgCl_2_, 0.9 mM CaCl_2_, 100 U/mL penicillin, and 100 μg/mL streptomycin; Life Technologies) at the indicated MOIs. Infections for multicycle propagation in MLE-15 cells were performed for 1 h at room temperature, and infections for single-cycle virus replication were performed for 1 h on ice. After the adsorption time, the inoculum was removed and monolayers were washed three times with PBS++. An appropriate amount of infection medium (DMEM supplemented with 0.2% [wt/vol] BSA and penicillin-streptomycin) was added, and cells were incubated for the indicated periods at 37°C. Infectious particles in the cell supernatants were quantified by foci assays on MDCK-II cells grown in 96-well plates.

### Indirect immunofluorescence.

293T cells were grown on coverslips in 24-well plates coated with poly-l-lysine. The transfected or infected cells were washed three times with PBS and fixed with 500 μL 4% (vol/vol) paraformaldehyde at room temperature for 8 min. After washing three times with PBS, cell membranes were permeabilized with 0.5% (vol/vol) Triton X-100, and unspecific antibody binding was blocked with blocking solution (PBS containing 10% [wt/vol] BSA and 0.5% [vol/vol] Triton X-100) at room temperature for 1 h. Cells were incubated with rabbit anti-M1, mouse anti-NP, mouse anti-M2, mouse anti-STRN, or mouse anti-STRN3 primary antibodies in PBS containing 1% (wt/vol) BSA and 0.5% (vol/vol) Triton X-100 at 4°C for 16 h. Cells were washed three times with PBS and incubated with Alexa Fluor 488- and Cy3-conjugated secondary antibodies for 1 h at room temperature. After incubation, cells were washed three times, and DNA was stained with 1 μg/mL Hoechst 33342. Coverslips were washed again and mounted on microscope slides with Mowiol mounting medium (Carl Roth). Slides were protected from light and stored at 4°C.

### Microscope image acquisition.

Confocal images were acquired with a Zeiss confocal LSM 710 microscope equipped with a Plan-Apochromat 63×/1.40 oil differential interference contrast M27 objective. Samples were illuminated using a diode (405-nm) laser, a DPSS (561-nm) laser, or an argon (488-nm) laser. Pictures were acquired and scale bars were calculated using the Zen Lite system (ZEN3.1, Blue edition). STRN3 superclusters were quantified for each image with prezoom of 0.6 at a frame size of 512 by 512 pixels, resulting in a resolution of 0.44 μm/pixel, and further analyzed in ImageJ. Threshold was set to 2× background intensity, and the “Analyze Particles” settings were size 0 to infinity, circularity 0.20 to 0.50. Fluorescence intensities were quantified with ImageJ and statistically analyzed using GraphPad Prism 9.

### Cell lysis and co-IPs.

To prepare cell extracts under native conditions for co-IPs, the cells were washed once with 1× PBS, harvested by scraping, and collected by centrifugation for 3 min at 300 × *g*. The cells were resuspended in an appropriate amount of IGEPAL lysis buffer (20 mM Tris-HCl [pH 7.4], 150 mM NaCl, 1% [vol/vol] IGEPAL CA-630 [Sigma], 10% [vol/vol] glycerol, 1 mM phenylmethylsulfonylfluoride, 10 mM NaF, 0.5 mM sodium orthovanadate, 1 μg/mL leupeptine, and 2 μg/mL aprotinin) and incubated on ice for 20 min. The lysates were cleared by centrifugation for 10 min at 4°C and 16,000 × *g*. The supernatant was then used for IP experiments or mixed with 5× SDS sample buffer, boiled at 95°C for 5 min, and analyzed by Western blotting as described elsewhere (71). Subcellular fractionation into the cytosolic, nuclear soluble, and chromatin fractions was performed as described previously (72). The IP was performed by adding 1 μg of anti-M1 or control IgG antibody to each sample in IGEPAL lysis buffer together with 25 μL protein A/G agarose beads (Millipore). After rotation for 4 h at 4°C, beads were washed five times with cold IGEPAL buffer and further processed for Western blotting or mass spectrometry.

### Mass spectrometry.

Samples bound to (magnetic) beads were washed three times with 100 μL 0.1 M ammonium bicarbonate solution. Proteins were digested “on-bead” by the addition of sequencing-grade modified trypsin (Serva) and incubated at 37°C for 45 min. Subsequently, the supernatant was transferred to fresh tubes and incubated at 37°C overnight. Peptides were desalted and concentrated using Chromabond C_18_WP spin columns (Macherey-Nagel catalog number 730522). Finally, peptides were dissolved in 25 μL of water with 5% (vol/vol) acetonitrile and 0.1% (vol/vol) formic acid. The mass spectrometric analysis of the samples was performed using a timsTOF Pro mass spectrometer (Bruker Daltonics). A nanoElute high-performance liquid chromatography system (Bruker Daltonics), equipped with an Aurora column (25-cm by 75-μm) C_18_ RP column filled with 1.7-μm beads (IonOpticks) was connected online to the mass spectrometer. A portion of approximately 200 ng of peptides corresponding to a 2-μL sample volume was injected directly on the separation column. Sample loading was performed at a constant pressure of 8 × 10^3^ Pa. Separation of the tryptic peptides was achieved at a 50°C column temperature with the following gradient of water–0.1% (vol/vol) formic acid (solvent A) and acetonitrile–0.1% (vol/vol) formic acid (solvent B) at a flow rate of 400 nL/min: linear increase from 2% (vol/vol) B to 17% (vol/vol) B within 18 min, followed by a linear gradient to 25% (vol/vol) B within 8 min and linear increase to 37% (vol/vol) solvent B in an additional 3 min. Data analysis was performed using MaxQuant (version 1.6.17.0) with the Andromeda search engine, and UniProt databases were used for annotating and assigning protein identifiers (73). Perseus software (version 1.6.14.0) was used for further analyses (74). Intensity values were log_2_ transformed. For the *t* test results and volcano plots in Fig. 4, 6, and 7, the missing values were imputed by 12, which was below the lowest measured value (with only one exception in all experiments) to allow statistical analysis.

### Analysis of M1 multimerization.

The 293T cells were transfected using the 8-plasmid reverse genetic system expressing SC35M M1 WT or M1 T108A/E mutants and incubated for 24 h. For the knockdown experiments, 293T cells were transfected twice with siRNAs 48 h apart. Two days after the second transfection, cells were further infected with SC35M virus (MOI = 3) on ice and incubated at 37°C for 6 h. Cells were washed three times in cold PBS and subsequently cross-linked by the addition of DSS or DSG at a final concentration of 2 mM. The reaction mixture was incubated for 30 min at room temperature. After removal of DSS or DSG, the cross-linking was quenched by the addition of 900 μL of PBS containing 20 mM Tris at room temperature for 15 min. Cells were then washed twice in PBS and lysed in IGEPAL buffer prior to SDS-PAGE.

### Polymerase reconstitution assay.

293T cells were transfected twice with siRNAs 48 h apart. Two days after the second transfection, cells were further transfected with pCAGGs plasmids encoding SC35M PB2, PB1, or PA (each 125 ng) or NP (800 ng) together with the firefly luciferase-encoding viral minigenome construct pHW72-Luci (25 ng) and a plasmid (pCI-neoRenilla-Luci; 20 ng). At 24 h.p.t., the cells were harvested, washed, lysed for 15 min in 150 μL of 1× passive lysis buffer. Firefly and *Renilla* luciferase activities were detected using the dual-luciferase reporter assay system (Promega) according to the manufacturer's protocol. *Renilla* activities were used for data normalization.

### Quantification and statistical analysis.

Image Lab 6.0.1 (Bio-Rad) and ImageJ software were used for image acquisition and densitometric analysis of Western blot data. GraphPad Prism 9 (GraphPad Software, La Jolla, CA, USA) was used to perform statistical analysis and generation of graphs. The statistical significance was calculated by using the unpaired Student's *t* test for two-group comparisons using the SPSS 19 program; *P* values of ≤0.05 were considered significant. Unless otherwise noted, error bars indicate the means and standard deviations of at least three independent experiments. Processing of confocal microscopy pictures and calculation of scale bars were done using Zen Lite software (ZEN3.1, Blue edition). Protein structures were analyzed using PyMOL (version 2.4.1.), and the STRING database (version 11.5) was used to reveal known and predicted interactions between the M1 interactors.

### Biosafety.

Viral infections were performed in accordance with German biosafety regulations pertaining to the propagation of IAVs, using biosafety level 3 containment laboratories approved for such use by the local authorities (RP, Giessen, Germany).
