# Supplementary material for: Phosphorylation of Influenza A Virus Matrix Protein 1 at Threonine 108 Controls Its Multimerization State and Functional Association with the STRIPAK Complex
Source: mBio. 2023 Jan 5;14(1):e03231-22. doi: 10.1128/mbio.03231-22 (PMC9973344; doi:10.1128/mbio.03231-22)
Supplement: FIG S1 [file mbio.03231-22-sf001.pdf]

Supplementary Figures

Lu Liu, Axel Weber, Uwe Linne, Mahmoud Shehata, Stephan Pleschka, Michael Kracht and M. Lienhard SCHMITZ

Phosphorylation of influenza matrix protein 1 at T108 controls its multimerization state and functional association with the STRIPAK complex

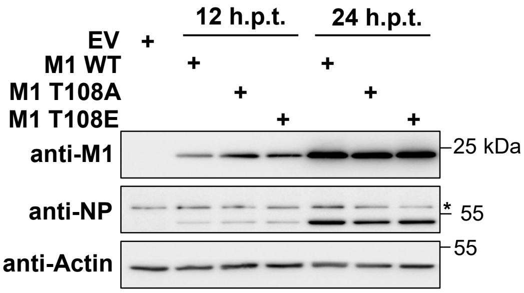

Suppl. Fig. S1. Substitutions at M1 T108 do not affect the M1 protein expression levels. 293T cells were transfected to express the SC35M genome together with WT M1 or its phosphorylation-defective or phospho-mimicking versions. Extracts from cells lysed at 12 and 24 h.p.t. were further analyzed by immunoblotting for protein expression as shown.
