# Supplementary material for: Phosphorylation of Influenza A Virus Matrix Protein 1 at Threonine 108 Controls Its Multimerization State and Functional Association with the STRIPAK Complex
Source: mBio. 2023 Jan 5;14(1):e03231-22. doi: 10.1128/mbio.03231-22 (PMC9973344; doi:10.1128/mbio.03231-22)
Supplement: FIG S2 [file mbio.03231-22-sf002.pdf]

Supplementary Figures

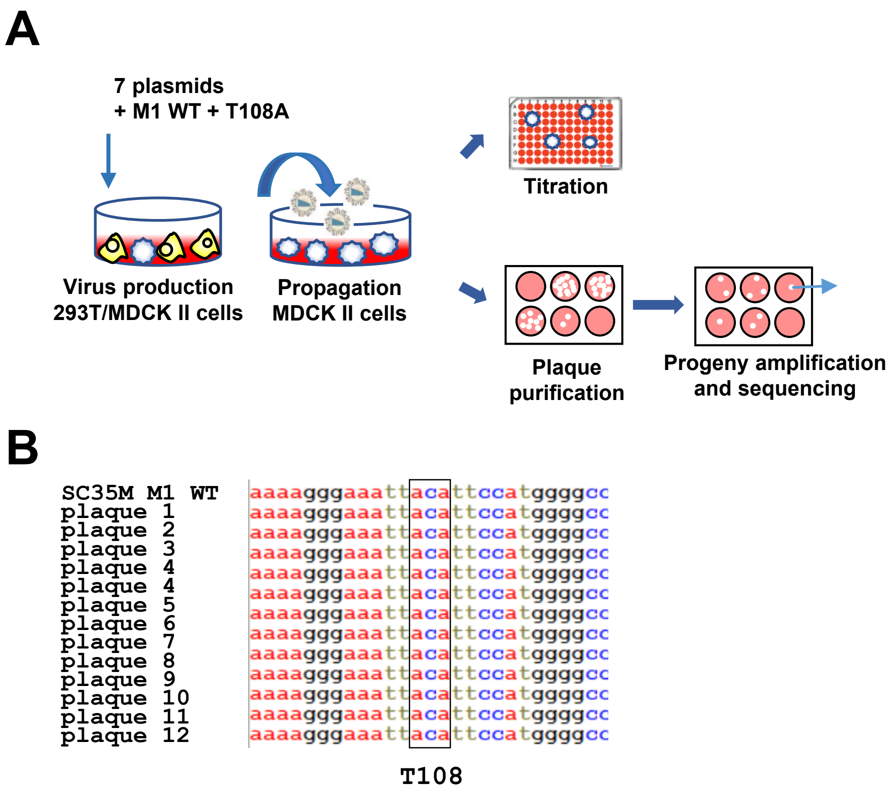

Suppl. Fig. S2. Rescue experiments. (A) 293T/MDCKII cells were transfected to express the SC35M genome using bidirectional pHW2000 plasmids in an approach cotransfecting M1 WT together with M1 T108A. IAVs contained in supernatants were further characterized by titration and virus progeny was isolated from individual plaques as shown schematically. Twelve plaques were purified, followed by amplification of the M1 gene using PCR and sequencing. **(B)** Results of the sequencing experiments are shown, the T108 codon is boxed.
