# Supplementary material for: Phosphorylation of Influenza A Virus Matrix Protein 1 at Threonine 108 Controls Its Multimerization State and Functional Association with the STRIPAK Complex
Source: mBio. 2023 Jan 5;14(1):e03231-22. doi: 10.1128/mbio.03231-22 (PMC9973344; doi:10.1128/mbio.03231-22)
Supplement: FIG S3 [file mbio.03231-22-sf003.pdf]

Supplementary Figures

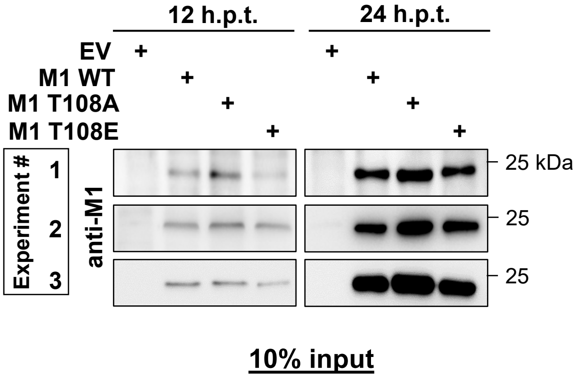

Suppl. Fig. S3. Quality check of M1 expression before analysis by mass spectrometry. 293T cells expressing 8 plasmids encoding SC35M together with M1 WT, M1 T108A or M1 T108E were lysed. While 90 % of the material was used for Co-IP and mass spectrometry as described in the legend for Fig. 4, the remaining material analyzed here was used for immunoblotting to ensure comparable and proper expression of M1. Results from all three biological replicates are shown.
