# Supplementary material for: Phosphorylation of Influenza A Virus Matrix Protein 1 at Threonine 108 Controls Its Multimerization State and Functional Association with the STRIPAK Complex
Source: mBio. 2023 Jan 5;14(1):e03231-22. doi: 10.1128/mbio.03231-22 (PMC9973344; doi:10.1128/mbio.03231-22)
Supplement: FIG S4 [file mbio.03231-22-sf004.pdf]

Supplementary Figures

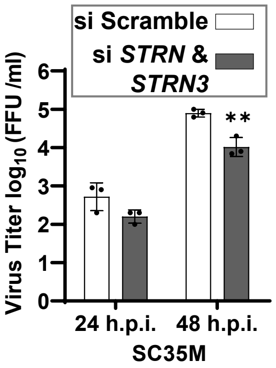

Suppl. Fig. S4. Effect of the STRN/STRN3 on SC35M replication in 293T cells. Expression of *STRN*/*STRN3* was reduced upon knockdown with specific siRNAs. Cells were infected with SC35M virus (MOI = 0.001) and the viral titers were determined at the indicated time points. Bars indicate means ± s.d. obtained from three independent experiments performed in triplicates, asterisks indicate *P* values (***P* ≤ 0.01).
