# Supplementary material for: Phosphorylation of Influenza A Virus Matrix Protein 1 at Threonine 108 Controls Its Multimerization State and Functional Association with the STRIPAK Complex
Source: mBio. 2023 Jan 5;14(1):e03231-22. doi: 10.1128/mbio.03231-22 (PMC9973344; doi:10.1128/mbio.03231-22)
Supplement: FIG S5 [file mbio.03231-22-sf005.pdf]

Supplementary Figures

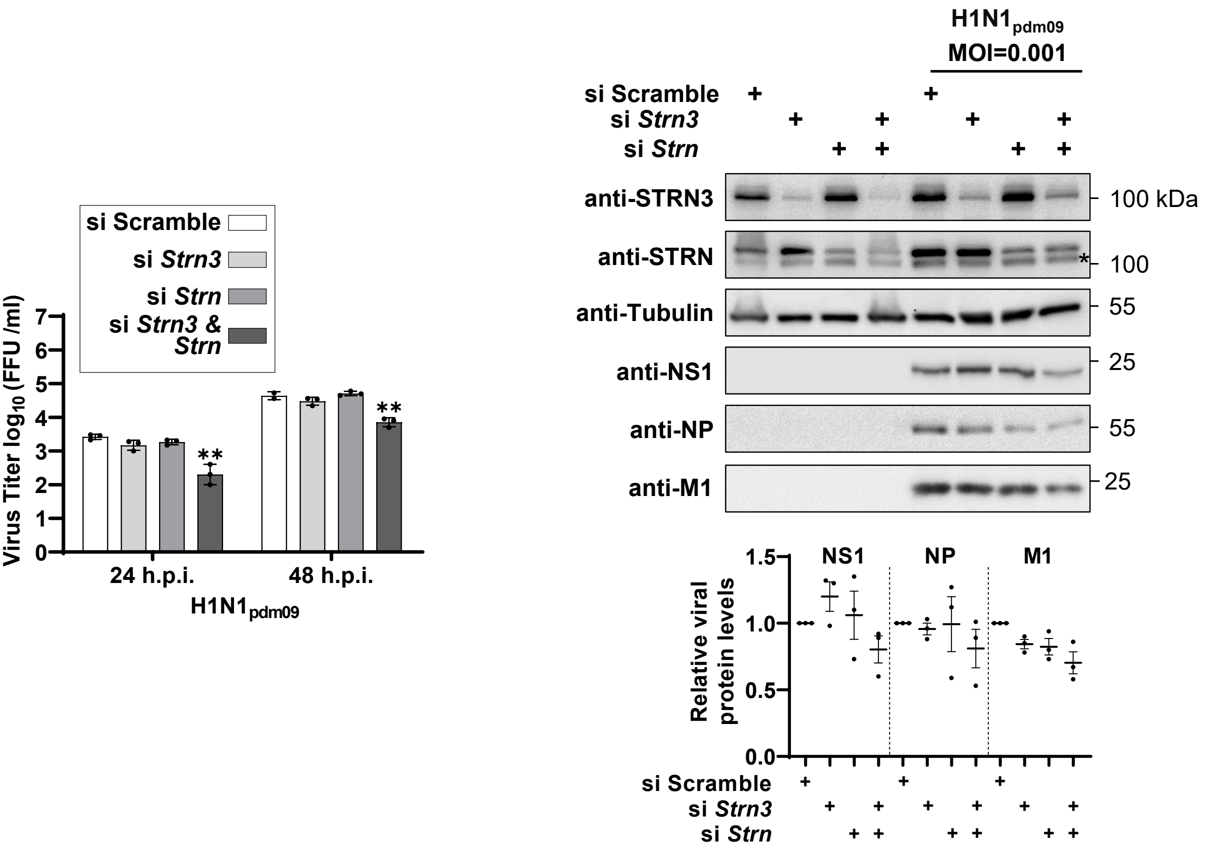

Suppl. Fig. S5. Effect of the Striatin complex on replication of H1N1$_{pdm09}$. MLE-15 cells were seeded and transfected one and three days later with siRNAs targeting mRNAs encoding *Strn*/*Strn3* or an adequate scrambled control as shown. Left: One fraction of the cells was infected with SC35M (MOI = 0.001) and viral titers of cell culture supernatants were determined at the indicated time points. Bars indicate means ± s.d. obtained from three independent experiments performed in triplicates by unpaired t-tests, asterisks indicate *P* values (**P* ≤ 0.05, ***P* ≤ 0.01). Right: The remaining fraction of the cells was lysed and tested by Western blotting for adequate knockdown and expression of viral proteins as shown, the position of a non-specific band is highlighted by an asterisk. The lower part shows a quantification of protein expression from three independent experiments, expression in the controls was set to 1, the means ± SEM are indicated.
