# Supplementary material for: Phosphorylation of Influenza A Virus Matrix Protein 1 at Threonine 108 Controls Its Multimerization State and Functional Association with the STRIPAK Complex
Source: mBio. 2023 Jan 5;14(1):e03231-22. doi: 10.1128/mbio.03231-22 (PMC9973344; doi:10.1128/mbio.03231-22)
Supplement: FIG S6 [file mbio.03231-22-sf006.pdf]

Supplementary Figures

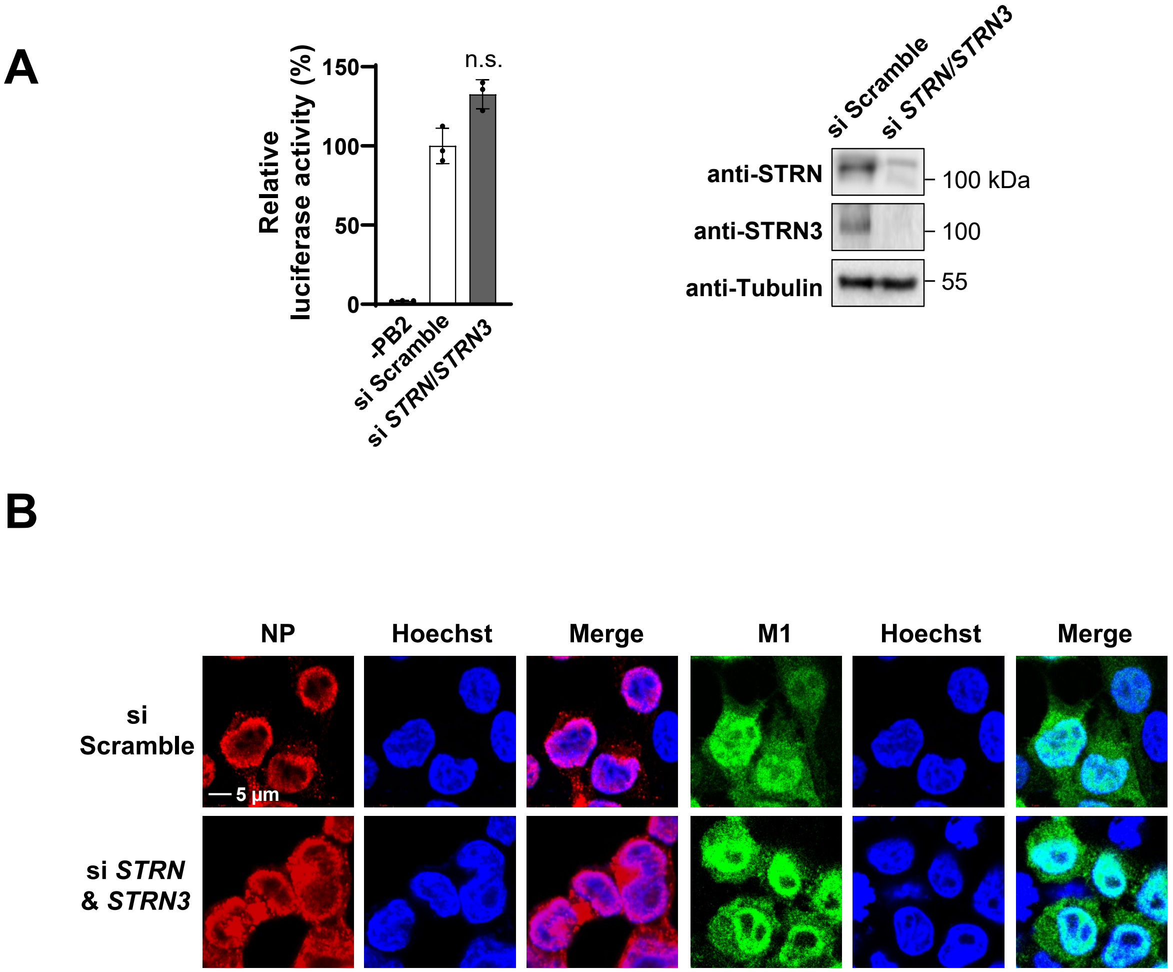

Suppl. Fig. S6. Effect of the STRN/STRN3 on polymerase activity and localization of viral proteins. (A) Expression of *STRN*/*STRN3* was reduced upon knockdown with specific siRNAs in 293T cells. Left: After knockdown the cells were transfected to express a Pol1- promoter driven vRNA-like transcript encoding the reporter gene together with pCAGGS plasmids allowing the expression of mRNAs encoding the indicated viral proteins. One day later, cells were harvested and activities from Firefly luciferase (generated by vRNP polymerase activity) and Renilla luciferase (used for normalization) were determined. Activity by the WT vRNP was set to 100%, error bars represent ± SEM from three independent experiments, n.s. = not significant. Right: Another part of the cells was analyzed by immunoblotting for successful knockdown as shown. **(B)** Another fraction of the siRNA-treated cells was infected by SC35M (MOI = 3) and intracellular localization of the indicated proteins was determined by indirect immunofluorescence as shown. Nuclei were stained with Hoechst 33342 (blue), pictures are representatives of two independent experiments.
