# Supplementary material for: Phosphorylation of Influenza A Virus Matrix Protein 1 at Threonine 108 Controls Its Multimerization State and Functional Association with the STRIPAK Complex
Source: mBio. 2023 Jan 5;14(1):e03231-22. doi: 10.1128/mbio.03231-22 (PMC9973344; doi:10.1128/mbio.03231-22)
Supplement: TABLE S4 [file mbio.03231-22-st004.docx]

**Supplementary Information**

**Supplementary Table 4**

**Antibodies**

| **Primary antibody (clone)** | **Species** | **Supplier** | **Cat.N** |
| --- | --- | --- | --- |
| anti-IAV M1(SC35M) | rabbit pAb | GeneTex | GTX125928 |
| anti-IAV M1(H1N1_pdm09_) | rabbit pAb | GeneTex | GTX127356 |
| anti-IAV NP | rabbit pAb | Thermo Scientific | PA5-32242 |
| anti-IAV NP | mouse mAb | Dr. S. Ludwig, Münster, Germany | |
| anti-IAV NS1 (SC35M) | mouse mAb |  |  |
| anti-IAV M2 (14C2) | mouse mAb | Thermo Scientific | MA1-082 |
| anti-STRN | mouse mAb | BD Biosciences | 610838 |
| anti-STRN3 | mouse mAb | Novus Bio | NB110-74572SS |
| anti- IAV NS1 (H1N1_pdm09_) | mouse mAb | Santa Cruz | SC-130568 |
| anti-PARP (C-2-10) | mouse mAb | Sigma | P248 |
| anti-Histone H3 | rabbit pAb | Abcam | ab1791 |
| anti-α-Tubulin (12G10) | mouse mAb | DSHB | 12G10 |
| anti-β-Actin | rabbit pAb | Abcam | ab8227 |
| anti-Vinculin | mouse mAb | Sigma | V9131 |
| anti-GAPDH (6C5) | mouse mAb | Abcam | ab8245 |
| IgG control | mouse pAb | Santa Cruz | SC-2025 |
| IgG control | rabbit pAb | Cell signaling | 2729S |

| **Secondary antibody** | **Conjugated to** | **Supplier** |
| --- | --- | --- |
| goat-anti-rabbit IgG | Alexa Fluor 488 | Dianova |
| goat-anti-mouse IgG | Cy3 | Dianova |
| goat-anti-mouse IgG | HRP | Dianova |
| goat-anti-rabbit IgG | HRP | Dianova |

**DNA Oligonucleotides**

| **Oligo name** | **Sequence (5´ to 3´)** |
| --- | --- |
| M1-T108A-fw | GCTCAAAAGGGAAATTGCATTCCATGGGGCCAAGGAGGTAC |
| M1-T108A -rv | CTTGGCCCCATGGAATGCAATTTCCCTTTTGAGCTTCCTGTAC |
| M1-T108E-fw | GCTCAAAAGGGAAATTGAATTCCATGGGGCCAAGGAGGTA |
| M1-T108E-rv | CTTGGCCCCATGGAATTCAATTTCCCTTTTGAGCTTCCTGTAC |
| One-Step RT-PCR (Universal Hoffman primers, PMID: 11811679) | |
| M1-fw | TATTCGTCTCAGGGAGCAAAAGCAGGTAG |
| M1-rv | ATATCGTCTCGTATTAGTAGAAACAAGGTAGTTTTT |

**siRNA Oligonucleotides**

| **Target** | **Sequence (Sense 5´to 3´)** | **Supplier** | **Catalog #** |
| --- | --- | --- | --- |
| *Strn* | SR420066A: ACGCUAGAAUCCAAUGUUGAUUCAA  SR420066B: AUACGCUUAUGGAAUUUAGAAAGTA  SR420066C: GAACAGUUGAUCAAUACUCUAUCTA | Origene Technology | SR420066 |
| *STRN*#1 | CUGCGUUUAUGGAAUACAAtt | Thermo Scientific | Assay ID s13587 |
| *STRN*#2 | CCUAGAAGCUGUUACAAGUtt | Thermo Scientific | Assay ID s13588 |
| *STRN3/Strn3* | CUUGCAGACUUGACGGUAAtt | Thermo Scientific | Assay ID s97012 |
| control scrambled siRNA | the sequence is undisclosed | Origene Technology | SR30004 |
| control scrambled siRNA | the sequence is undisclosed | Thermo Scientific | D-001810-10-05 |

**Plasmids**

| **Plasmid** | **Reference** |
| --- | --- |
| pHW2000 plasmids encoding SC35M segments | PMID: 16339318 |
| pHW2000-SC35M-M-T108A | this study |
| pHW2000-SC35M-M-T108E | this study |
| pCAGGS plasmids coding for SC35M PB2, PB1, PA, NP proteins | PMID: 21183679 |
| pHW72-Luci | PMID: 18053252 |
| pCI-neoRenilla-Luci | PMID: 28615693, PMID: 16081698 |
| pcDNA3.1-Striatin-HA | PMID: 30592649 |
| pMet7-Flag-STRN3 | PMID: 28827617 |
